# Embodied Conversational Agents for Patients With Dementia: Thematic Literature Analysis

**DOI:** 10.2196/25381

**Published:** 2021-07-16

**Authors:** Margherita Rampioni, Vera Stara, Elisa Felici, Lorena Rossi, Susy Paolini

**Affiliations:** 1 Model of Care and New Technologies IRCCS INRCA - National Institute of Health and Science on Aging Ancona Italy; 2 Unit of Neurology IRCCS INRCA - National Institute of Health and Science on Aging Ancona Italy

**Keywords:** dementia, patient with dementia, older adults with dementia, embodied conversational agent, virtual personal assistant, virtual agent, virtual companion, design for older adults, patients, elderly, virtual, personal assistant, cognitive, cognitive impairment

## Abstract

**Background:**

As the world’s population rapidly ages, the number of older adults with cognitive impairment will also increase. Several studies have identified numerous complex needs of people with dementia, which assistive technologies still fail to support. Recent trends have led to an increasing focus on the use of embodied conversational agents (ECAs) as virtual entities able to interact with a person through natural and familiar verbal and nonverbal communication. The use of ECAs could improve the accessibility and acceptance of assistive technologies matching those high-level needs that are not well covered to date.

**Objective:**

The aim of this thematic literature analysis was to map current studies in the field of designing ECAs for patients with dementia in order to identify the existing research trend and possible gaps that need to be covered in the near future. The review questions in this study were as follows: (1) what research frameworks are used to study the interaction between patients with dementia and ECAs? (2) what are the findings? and (3) what are the barriers reported in these studies?

**Methods:**

Separate literature searches were conducted in PubMed, Web of Science, Scopus, and Embase databases by using specific umbrella phrases to target the population (patients with dementia) and the technology-based intervention (embodied conversational agent). Studies that met the inclusion criteria were appraised through the Mixed Methods Appraisal Tool and then discussed in a thematic analysis.

**Results:**

The search process identified 115 records from the databases and study references. After duplicates (n=45) were removed, 70 papers remained for the initial screening. A total of 7 studies were finally included in the qualitative synthesis. A thematic analysis of the reviewed studies identified major themes and subthemes: the research frameworks used to gather users’ perspectives on ECAs (theme 1), the insights shared by the 7 studies as well as the value of user involvement in the development phases and the challenge of matching the system functionalities with the users’ needs (theme 2), and the main methodological and technical problems faced by each study team (theme 3).

**Conclusions:**

Our thematic literature analysis shows that the field of ECAs is novel and poorly discussed in the scientific community and that more sophisticated study designs and proofs of efficacy of the approach are required. Therefore, by analyzing the main topic of the narrative review, this study underscores the challenge of synchronizing and harmonizing knowledge, efforts, and challenges in the dementia care field and its person-centered paradigm through the user-centered design approach. Enabling strict collaboration between interdisciplinary research networks, medical scientists, technology developers, patients, and their formal and informal caregivers is still a great challenge in the field of technologies for older adults.

## Introduction

### Background

The world’s population is rapidly aging and approximately 47 million people are now experiencing dementia worldwide. This number could triple by 2050 with an incremental estimated cost that will range from US $818 billion in 2015 to US $2 trillion by 2030 [1]. Dementia is characterized by the progressive deterioration of both cognitive and functional abilities that affect a person’s capability to perform everyday activities [2,3]. Nowadays, the tendency to view dementia solely within a medical framework is overcome through a new personhood paradigm based on the identification of the numerous complex needs of patients with dementia as described by Maslow [4] and Kitwood [5]. There is consensus that individuals living independently [6] or in long-term care [7-9] are able to express needs [10] and preferences [11] consistently, even in the advanced stage of dementia [12].

Since the challenges of responding to the growing number of people with dementia and their complex needs are substantial for governments [13], the field of assistive technologies is receiving more and more interest. In aged care, the term “assistive technology” refers to any device, product, or equipment that helps people to perform a task they would otherwise be unable to do or that facilitates older adults’ activities of daily living [14]. In dementia care, assistive technologies (ie, technologies for daily living, meaningful and pleasurable activities and health care) can compensate for cognitive impairment, improve quality of life, favor autonomy, enable people to remain in their homes for longer, and reduce care costs [15-19]. Actually, a broad spectrum of technologies supports community-dwelling persons with dementia. These technologies mostly address basic physiological and safety needs, whereas little attention is devoted to higher-level needs such as self-esteem, quality of life, recreational activities or contrast behavioral issues, for example, aggression and mood changes [6,20-22]. Recently, several studies [23-27] proposed the use of screen-based entities designed to stimulate human face-to-face conversation skills called as embodied conversational agents (ECAs) or personal virtual assistants [28,29]. Such virtual entities are able to interact with a person through verbal and nonverbal communication. There is a significant and growing list of use cases for ECAs targeting older adults with or without cognitive impairment or their caregivers [15]: virtual coaches [30], virtual companions [31-33], personal virtual assistants [26], virtual butlers [34,35], and training tools to help formal and informal caregivers [36].

The use of ECAs could improve the accessibility and acceptance of computer-based assistive technologies when compared to graphical user and voice interfaces, especially for older adults with cognitive impairment [37-39], thus matching those high-level needs that are not satisfactorily covered by assistive technologies. The extent to which this specific innovation may be able to support people affected by dementia along the progressive nature of the disease represents a great challenge for the entire scientific community. Unfortunately, it seems that research in that specific direction is still poor and little is known on how patients with dementia interact with ECAs and how this interaction should be designed and managed by the system [15]. Therefore, to bridge this gap, this paper discusses the implications derived from a thematic literature review of the available studies focusing on personal virtual assistants in favor of patients with dementia.

### Aim of This Study

The aim of this thematic literature analysis was to map current studies in the field of designing ECAs for patients with dementia in order to identify the existing research trends and possible gaps that need to be covered in the near future. The review questions were (1) what research frameworks are used to study the interaction between patients with dementia and ECAs? (2) what are the findings? and (3) what are the barriers reported in these studies?

## Methods

### Design of This Study

**Figure 1:**
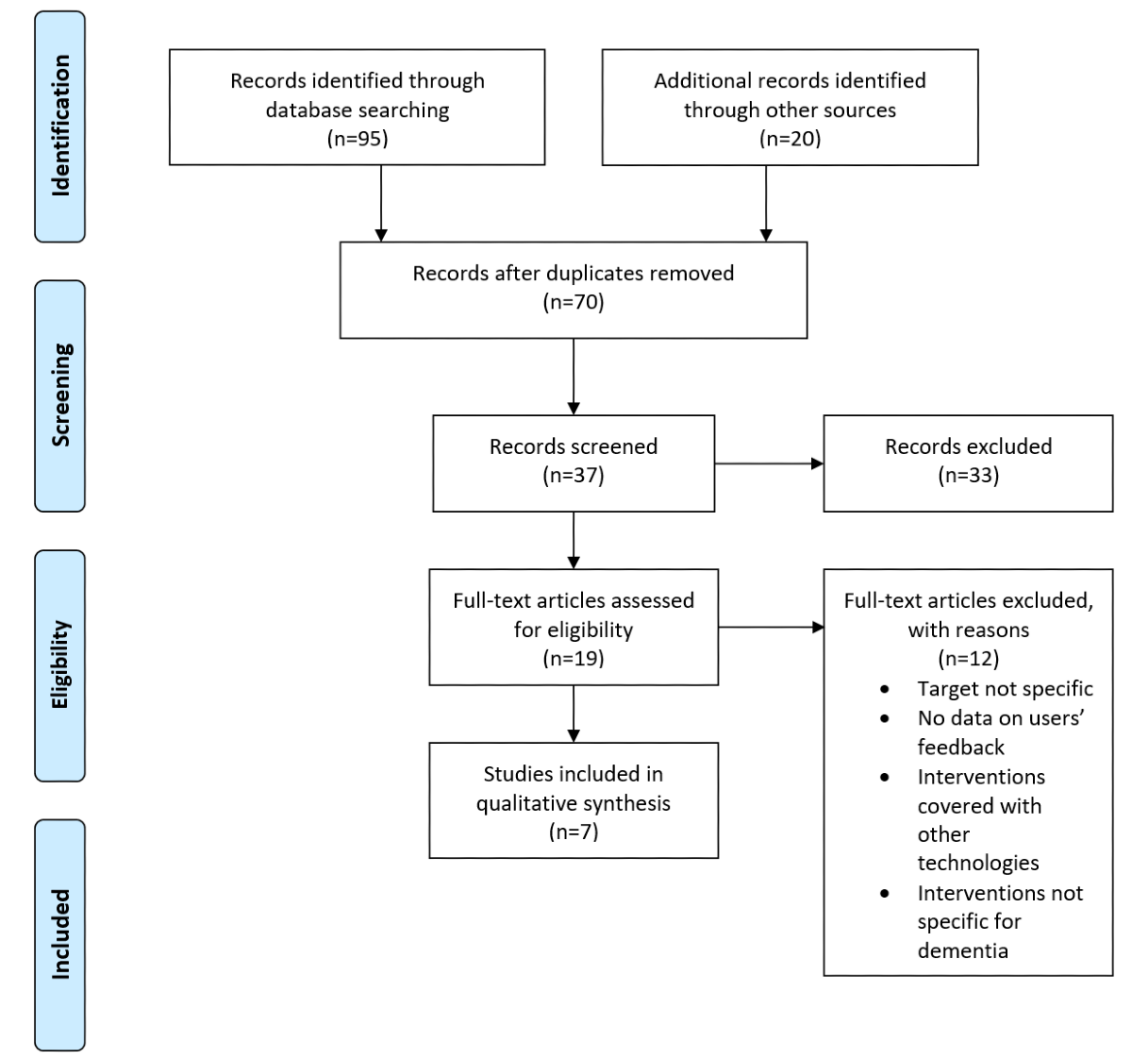
Flow diagram of the studies included in the thematic review as well as the main reasons for rejection.

Separate literature searches were conducted in PubMed, Web of Science, Scopus, and Embase databases by using the following umbrella phrases to target the population and the specific technology-based intervention: (“patient with dementia” OR “people with dementia” OR “person with dementia”) AND (“virtual agent” OR “personal virtual assistant” OR “virtual companion” OR “embodied conversational agent”). Inclusion criteria were published papers written in English with the aim of studying the use of ECAs (1) among older adults (≥65 years) with dementia living at home, in long-term care, or nursing homes and their formal and informal caregivers, (2) for coping in patients with dementia without any restriction in terms of service applications (ie, cognitive games, reminders, medicine intake, calendar, etc), (3) for presenting empirical findings about interactions between users and ECAs, and (4) in randomized controlled trials (qualitative, quantitative, and the mixed methods approach were included). There was no restriction on publication dates, and the searches were finalized in July 2020. Papers were excluded if reviews, theoretical or technical studies, and contributions were not original research papers that met the inclusion criteria or were not written in English. According to predefined criteria, the screening phase was based on the analysis of titles and then abstracts. Later, full texts of those titles/abstracts of screened publications were reviewed independently by the first and the corresponding author in August 2020. Another researcher was involved in reaching consensus in cases of disagreement. Studies that met the inclusion criteria were included, and the results of the searches were summarized. Then, we performed a manual thematic analysis of the findings. We used the Preferred Reporting Items for Systematic Reviews and Meta-Analysis [40] flowchart in the retrieval and selection process (Figure 1, Multimedia Appendix 1).

### Quality Appraisal

Three authors independently appraised the final papers for their methodological quality by using the Mixed Methods Appraisal Tool version 2018 [41]. The Mixed Methods Appraisal Tool assesses the quality of qualitative, quantitative, and mixed methods studies and can be used to appraise the quality of different study designs. Precisely, it focuses on the methodological criteria and includes 5 core quality criteria for qualitative, randomized controlled, nonrandomized, quantitative descriptive, and mixed methods. Owing to these advantages, it was chosen over other tools prior to starting the narrative review. The results of each appraisal were compared, and any disagreement was solved through the intervention of the last author and discussion among the authors. According to the scoring system proposed by Pluye et al [42], a quantitative appraisal score was calculated by assessing the presence/absence of criteria (yes/no). The quality score was calculated as a percentage by using the formula: (number of “yes” responses divided by the number of appropriate criteria) × 100.

### Thematic Analysis

A thematic analysis was conducted to identify themes based on the 6 phases described by Braun and Clarke [43]. Themes were coded based on our specific review questions by following these steps: first, authors read the data from each study; and second, the corresponding author carried out a systematic manual coding of the features that led to initial codes before searching for themes in the third step. Themes were then reviewed for correlation with codes and the identification of subthemes during the fourth phase. After defining the themes in phase five, findings were evaluated for relevance to the research questions. Finally, the authors discussed the analysis process and reached consensus on the labelling.

## Results

### Reviewed Studies

The search process identified 95 records from the databases and an additional 20 by manually searching those studies’ references. After duplicates (n=45) were removed, 70 papers remained for initial screening by title. This resulted in 37 potentially eligible abstracts. The abstracts retained were analyzed by authors according to the research questions in order to obtain the final list of full-text papers to be reviewed. After the analysis of the abstracts, 33 of them were excluded as they did not fit the settled criteria of the target population and the specific technology-based intervention. A second screening step was performed for those full-text papers that matched all the criteria (n=19). From this process, 12 papers were excluded because (1) studies recruited both patients with dementia and older adults in good health status (n=3), (2) no empirical feedback from users was reported (n=3), (3) the intervention was performed using both ECAs and other technologies (n=2), and (4) the intervention was not specific for patients with dementia (n=4). Finally, a total of 7 studies were included.

A summary of the studies and their findings are presented in Table 1. The 7 studies were conducted in Spain [24], Japan [27], France [15,44], Canada [45], Italy, and Luxembourg [46,47] and published between 2008 and 2019. They were heterogeneous in terms of objectives, population, contexts, and methodologies. Four of the 7 studies describe the development of 2 specific agents: Louise [15,44] and Anne [46,47].

**Figure 2:**
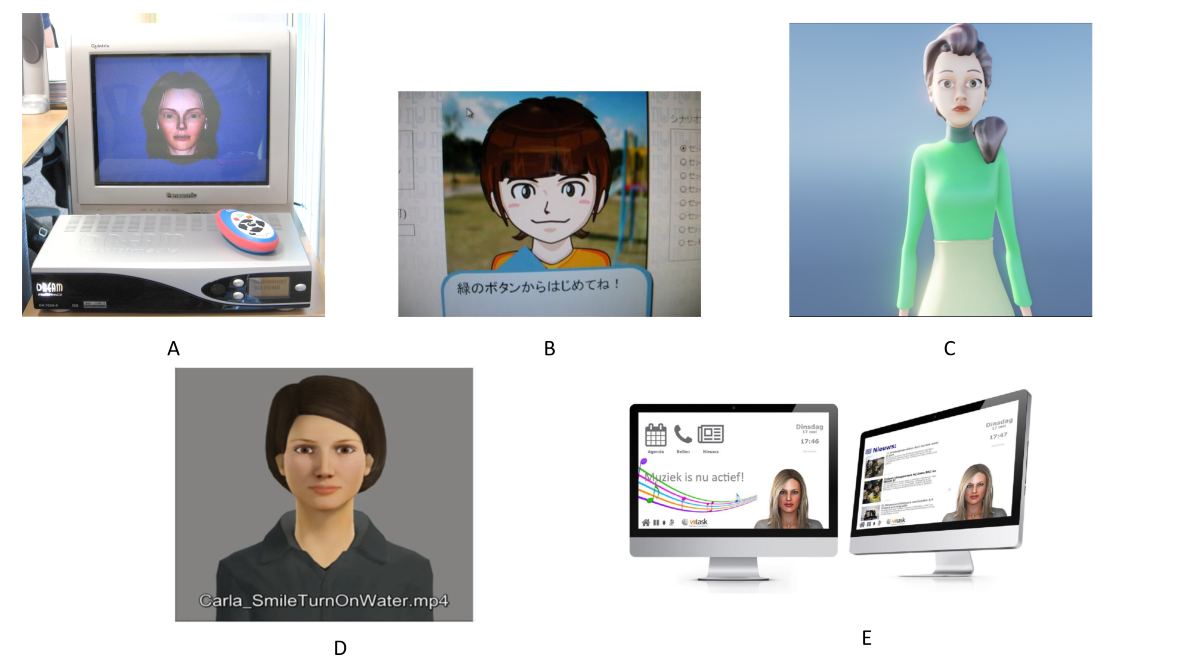
Embodied conversational agents described in the reported studies. A) Carrasco et al [24]; B) Yasuda et al [27]; C) Wargnier et al [15,44]; D) Konig et al [45]; E) de Jong et al [46] and Stara et al [47].

The different agents are shown in Figure 2. Carrasco et al [24] built a prototype that included a tool for real-time streaming of a realistic avatar, previously programmed by a caregiver, to a television. This technique was used to simulate a true virtual assistant on the television screen. The avatar has a realistic voice and the lips are in synchronization with its speech to ensure that its facial movements appear natural. Yasuda et al [27] developed an agent conversation system shown on a computer screen in the form of an animated face resembling a 5-year-old grandchild. This system can detect the end of the speech sound of a subject’s reply to a question and begins asking the next question. When the subject speaks, the agent reacts by automatically generating nods, mouth movements, and acknowledgments. Wargnier et al [15,44] proposed a prototype of a semiautomatic system that allows the animation of a female cartoon-like character called Louise and speech synthesis from text. The character, displayed on either a computer screen or a television set, is in an idle pose and moves its lips while speaking. This system includes attention monitoring and interaction management based on a predefined script and keyboard inputs of a (presumably hidden) operator. Konig et al [45] developed an emotionally intelligent cognitive assistant in the form of a humanoid female character that shows up on a screen in an inactive pose. de Jong et al [46] and Stara et al [47] shared the know-how gained in the design and adaptation of the personal virtual assistant Anne, which is a friendly, female human-looking avatar talking and interacting with users on a screen. Older adults can communicate with the ECA through voice and touch. This system is able to learn autonomously from its users and gets to know their personal preferences and needs.

**Table 1:** Summary of the reviewed studies.

Studies	Purpose	Type of system	Method for data collection	Sample	Country, test setting	Findings
Carrasco et al [24]	To validate a functional prototype that gives a measure on how natural the interaction between avatars and people with Alzheimer disease is. To increase the acceptability of the system by target users	Avatar displayed on a standard television set	Yes/No questions and one-to-one observation	21 persons had Alzheimer disease, with a Global Deterioration Scale [48] measure ranging from 3 to 5 (from mild to moderate)	Spain, day care center	All users engaged naturally with the avatar, understood the information conveyed by the avatar, and answered successfully by means of the television remote control
Yasuda et al [27]	To investigate the effectiveness of a conversation system based on an animated face of a child	A computer screen that shows an animated face of a child agent	Qualitative interviews	8 older adults (2 males and 6 females) had mild Alzheimer disease, with a Mini-Mental State Examination [49] mean score of 22.2. The average age was 78.5 years.	Japan, hospital	All users conversed with the conversational agent system and enjoyed the conversation.
Wargnier et al [44]	To collect design guidelines to develop a semiautomated ECA^a^ prototype	A semiautomated cartoon like ECA prototype that runs on a standard personal computer with Microsoft Windows	Semiautomated Wizard of Oz, video, observation, open interview, questionnaire	14 specialists (4 males and 10 females) in assistive technologies for older adults or care professionals (medical doctors and neuropsychologists, mostly)	France, hospital	All participants interacted naturally with the ECA. Most displayed high levels of attention. Globally, the feedbacks turned out to be rather positive.
Konig et al [45]	To identify affective identities in patients with dementia for the design of cognitive assistive technologies	An intelligent cognitive assistant in the form of a humanoid female character shown on a screen	Qualitative interview	12 older adult care home residents (5 males and 7 females) with Alzheimer disease who showed cognitive and functional impairment to an extent that it affected their autonomy in performing certain complex activities of daily living and 9 associated caregivers (2 males and 7 females). The average age of the residents was 84.5 years.	Canada, University and Research Institute for Aging	Definition of user requirements for the design
Wargnier et al [15]	To conduct a usability study to refine and validate the Louise ECA	A semiautomated cartoon like ECA prototype that runs on a standard personal computer under Microsoft Windows	Realistic assistive scenarios and semistructured interview	14 participants (3 males and 11 females) with mild cognitive impairment (9/14) or Alzheimer disease (5/14), whose Mini-Mental State Examination [49] scores ranged from 8 to 30 (mean 23.8, SD 4.9). The average age was 78.8 years.	France, hospital and University	Most of the participants were able to interact with the ECA, succeeded in completing the proposed tasks, and enjoyed the design
de Jong et al [46]	Report the first iteration of a comprehensive user-centered development process of virtual agents for patients with dementia and their caregivers	A personal assistant called Anne that works on a Surface Pro tablet under the Microsoft Windows 10 operating system	Focus group	16 caregivers: 10 in Luxembourg (6 qualified nursing assistants and 4 informal carers) and 6 in Italy (3 care professionals and 3 informal caregivers)	Luxembourg, Italy, hospital, day care center	Definition of user requirements for the design
Stara et al [47]	How patients xperience a personal virtual assistant in the stage of moderate dementia; how a personal virtual assistant can be modified to the requirements of people in the stage of moderate dementia	A personal assistant called Anne that works on a Surface Pro tablet under the Microsoft Windows 10 operating system	Protected environment test scenarios with observation of the interactions between patients and the personal virtual assistant and interview to formal caregivers	5 female patients with moderate dementia and 2 formal caregivers in Italy; 1 female patient with dementia and 2 formal caregivers in Luxembourg	Italy, Luxembourg, hospital, day care center	Definition of user requirements for the design

^a^ECA: embodied conversational agent.

### Quality Appraisal of the Selected Studies

The design of the 7 research studies was assessed by using screening questions and the 5 criteria of the Mixed Methods Appraisal Tool [41] for qualitative and mixed methods studies reported in Textbox 1: (1) appropriateness of objective/research question, (2) adequacy of the qualitative approach/method, (3) adequate gathering of findings from data, (4) sufficient interpretation of results from data, and (5) coherence between qualitative data sources, collection, analysis, and interpretation.

**Mixed Methods Appraisal Tool version 2018 criteria to appraise the study design.**

**Methodological quality criteria**

**Screening questions (for all types)**

S1. Are there clear research questions?

S2. Do the collected data allow to address the research questions?

**Qualitative**

Q1. Is the qualitative approach appropriate to answer the research question?

Q2. Are the qualitative data collection methods adequate to address the research question?

Q3. Are the findings adequately derived from the data?

Q4. Is the interpretation of results sufficiently substantiated by data?

Q5. Is there coherence between qualitative data sources, collection, analysis, and interpretation?

**Mixed methods**

M1. Is there an adequate rationale for using a mixed methods design to address the research question?

M2. Are the different components of the study effectively integrated to answer the research question?

M3. Are the outputs of the integration of qualitative and quantitative components adequately interpreted?

M4. Are divergences and inconsistencies between quantitative and qualitative results adequately addressed?

M5. Do the different components of the study adhere to the quality criteria of each tradition of the methods involved?

All the studies used appropriate research design, taking into account the research questions and problems related to the use of a specific innovation technology among persons with dementia. The prevalent data collection methods were open or semistructured interviews [15,27,45,47], observations [24,44,47], a questionnaire [24,44], and a focus group [46]. The findings and interpretations of the results were coherent with data sources, collection, analysis, and discussion in all the studies. According to Pluye et al [42], the score for each study was calculated and then synthetized using 3 different categories: low score, <35%; medium score, 36%-70%; and high score, 71%-100%. All studies met the 5 quality criteria; therefore, the score calculation synthesized a high score of methodological quality results (Table 2).

**Table 2:** Quality scores of the selected studies.

Studies	Screening question score	Qualitative studies score	Mixed methods studies score	Total score	Appropriate criteria (n)	Quantity score (%)	Score category
Carrasco et al [24]	2	N/A^a^	5	7	7	100	High
Yasuda et al [27]	2	5	N/A	7	7	100	High
Wargnier et al [44]	2	N/A	5	7	7	100	High
Konig et al [45]	2	5	N/A	7	7	100	High
Wargnier et al [15]	2	N/A	5	7	7	100	High
de Jong et al [46]	2	5	N/A	7	7	100	High
Stara et al [47]	2	5	N/A	7	7	100	High

^a^N/A: not applicable.

### Thematic Analysis

Following the analysis of reviewed studies, 3 major themes and subthemes within each theme were identified: (1) research frameworks, (2) efficacy of ECAs, (3) limitations of the studies and problems faced.

#### Theme 1: Research Frameworks

All the studies dealt with 2 research questions: (1) could virtual agent be a technology that patients with dementia can really use? and (2) which are the design features that can facilitate or hinder this usage? Methodologically, both qualitative and quantitative designs were applied to answer these questions focusing on meanings and understanding of experiences of people with dementia and their carers. In all cases, participants took part in the researches in participatory sessions or observations to avoid the discomfort of being the subjects of an experimental study. Overall, the 7 studies came to the general assumption that the use of ECAs is suitable for people with dementia. This common evidence based its foundation on the use of voice as an interaction modality between the systems. The voice as input/output modality is the natural and familiar way to engage people with dementia in such researches. Therefore, from 2008 to 2019, even though the readiness level of the virtual agents was changed considerably, especially in the human-looking appearance, none of the studies shared skepticism or disadvantages in the use of ECAs by the enrolled patients. Additionally, the use of common screen devices as the presentation platform on the television [24], computer [15,27,44,45], or tablet [47] concur with the positive engagement of users. A notable consideration that emphasizes this outcome is the rigorous recruitment strategy followed by each team: as reported in Table 1, enrolled participants were previously diagnosed and scored on the Global Deterioration Scale [48] and the Mini-Mental State Examination [49]. Moreover, the experimental settings were always under the responsibility of researchers or formal caregivers in all the studies. However, the objectives of the 7 studies were different from the research question about which feature design could improve or impede the use of virtual characters. This is due, in part, to the specific purpose of each system under development that varied from natural interaction [24,27,45] and their specificities [15,44] to support independent living [46,47]. Features such as interaction paradigm and prompting style are seen as the main components that could be personalized and used for matching the needs and capabilities of users, thereby improving the user experience. Probably because the ECA field is still green, none of the studies pursued the general purpose to achieve general features design as the guidelines for future researches in the field.

#### Theme 2: Efficacy of ECAs

##### Findings of the Selected Studies

As previously mentioned, all the studies reported positive feedback on the use of agents by users: the majority of patients with dementia naturally interacted and responded to the virtual character fulfilling the assigned tasks [15,24,27,45]. The artificial assistant caught the attention of people, and in some cases, it was seen as a companion by older participants as well as formal and informal caregivers [46,47]. On the contrary, the preference of having a “real person helping me rather than a machine” [45] clearly showed the importance of a human contact. Moreover, participants with the most severe memory impairment frequently forgot how to interact with the system or talk to the ECA and it caused them some frustration or they seemed to be intimidated by the character [15]. These results are in line with the findings of the study conducted by Stara et al [47], who found that patients with moderate dementia spoke freely to the ECA without using the touch control. Persons with dementia generally had little experience with mobile technology before they became affected by dementia; therefore, patients seemed to have trouble using a touchscreen and navigating the different applications of the ECA. For that reason, formal caregivers involved in this research suggested a supervised usage of the virtual agent in a controlled environment such as daily centers. Formal caregivers were strongly convinced that their patients with moderate dementia could not use Anne independently at home. For them, ECAs could be useful tools for their daily working activities but only if controlled by professional staff. Nonetheless, positive results in terms of efficacy are reported in studies from the perspective of formal caregivers [44,46,47]. In particular, according to de Jong et al [46], virtual agents could help and support people with forgetful problems who are living independently in their homes.

##### Value of a User-Driven Approach

User-centered design as “a philosophy based on the needs and interests of the user, with an emphasis on making products usable and understandable” [50] is a common methodology involving end users during all iterations of the design process [51]. Four of the reviewed studies dealt with the development of the ECA through this user-driven approach: Louise [15,44] and Anne [46,47]. Louise was developed by adopting a co-design living lab approach involving older adults with dementia and stakeholders such as care professionals. The ECA called Anne [46,47] was developed using the umbrella framework of the ISO standard user-centered design [51] and providing a human-centered perspective through the active involvement of patients and their formal and informal caregivers. For us, this underlies key strengths to overcome the main barriers in applying technology for older adults in general and, in particular, for people who experience dementia. Therefore, Louise and Anne could be considered as good examples for the next generations of ECAs that will benefit from user-centered design by using input from users, patients, formal and informal caregivers as well as clinicians and other stakeholders. As reported by de Jong et al [46] and Konig et al [45], examining feedback from the point of view of family caregivers or other carers (ie, nurses, health care professionals, or care workers who work with persons with dementia) could build more awareness on how to develop effective technologies. This benefit can create better design, thus enhancing usability, user experience, acceptance, and potential market success. Nonetheless, how users come to accept and use the given technology was mainly explored by Wargnier et al [15] and Stara et al [47] who evaluated Louise’s and Anne’s usability and acceptance through direct observations. In addition, Carrasco et al [24] observed users during their interactions with the system and noted their observations in usability reports. None of the other studies reported in this narrative review mentioned information on the user-driven approach despite all the studies being clearly oriented to define the requirements definitions of the ECAs as a starting point of the development process. These requirements were analyzed to map functionalities and preferences for the next iterations. The user and technical requirements definitions are indeed the first actions of the user-centered design process [51].

##### Matching Needs With Technology

People with dementia have many changing needs during the progression of their disease, varying from memory support to almost all aspects of daily functioning. In these studies, specific ECA functions matched the physiological, comfort, and attachment needs. ECAs described in 3 of the included studies [24,27,45] sought mainly to overcome the memory problems of persons with dementia, guide them in their daily activities, and meet their need for communication and social interaction. Memory problems are certainly a basic component of dementia, but communication, emotional, and behavioral problems represent significant issues too. The evolution of this disease can easily lead these people to refuse or forget to drink, eat, and take medication and to feel more alone, apathetic, and isolated. In these cases, the ECA could play a supporting role not only in helping patients to perform their daily activities more independently but also in stimulating dialogues and encouraging their participation in the conversation and, consequently, in maintaining social relationships. For example, in the study of Yasuda et al [27], a man with early onset dementia said, “In this system, I can talk freely without any hesitation or anxiety.” This comment means that conversations with normal people were stressful to him owing to the difficulty of answering questions that he cannot reply to. As dementia severity progresses, depression and boredom increase as well as the sense of powerlessness, lack of control, and social withdrawal. For this reason, 4 of the reviewed studies [15,44,46,47] designed ECAs to meet multiple and more complex user needs. More precisely, in the studies on Louise [15,44] and Anne [46,47], the development of multi-purpose tools is described. The virtual agent Anne [46,47] offers some features such as reminder (personal and medication agenda), communication (video calls), information (news), and entertainment (games and music) that support users in all aspects of daily life. In particular, these features engage persons with dementia in various activities and help them pass the time in a more meaningful way and improve their quality of life. Louise [15,44] proposes others features to patients such as guiding through a task, cognitive stimulation exercises, and attention management. In this latter case, the ECA aimed to compensate for users’ attentional disorders by performing autonomous prompting (ie, calling the user to regain attention in case of distractions). In summary, these multi-purpose agents are able to engage persons with dementia both emotionally and motivationally and stimulate their attentional skills, thereby making them feel part of a larger project and therefore less apathetic and isolated. This engagement could increase the physical, cognitive, and emotional well-being of patients but this possibility is only postulated in the mapped studies.

#### Theme 3: Limitations of These Studies and the Problems Faced

Common limitations and problems are reported in the selected studies. The challenge of small samples is clearly mentioned even if they underwent the preliminary evaluation of the systems [15,27,47]. This limitation is strictly correlated to the willingness to involve the entire spectrum of dementia severity [15,46,47] in order to adapt the virtual agents to the changing needs of older patients through various stages of this illness. A problem that arose concerns the importance of personalized solutions [47]. For example, a specific personalized prompting style should be programmed reaffirming the user in his or her overall persona and situational identity [45]. This means that each user has different preferences that may influence adherence to the system. For instance, people who want the ECA to address them in a very polite manner may reject the ECA considering it disrespectful if it calls them by their first name and uses a personal pronoun [15]. In addition, with regard to pleasantness, in the study of Wargnier et al [44], all users expressed a positive or very positive opinion and more than half (7/13) said they would like to be able to personalize the character’s appearance. Moreover, the ECA’s prompting styles is another feature that needs to be adapted since patients with neutral valence and weaker identity profiles prefer to be less in control and, therefore, did not mind a dominating prompting style. On the other hand, users with identities that were positive and powerful reported they prefer to be in control over what happens to them, thus preferring more subtle prompts [45].

## Discussion

### Principal Findings

This review surveyed the literature on the usage of ECAs by patients with dementia with the aim to identify the current research trends and possible gaps to cover in the future. Three main questions piloted this study: (1) what research frameworks are used to study the interaction between persons with dementia and ECAs? (2) what are the findings? and (3) what are the barriers? Only 7 papers were returned from the search. Overall, the main findings of this narrative review demonstrate that research on ECAs as an innovative way to cope with dementia is little covered in the state of the art even though interesting topics emerged from the mapped studies: the research design used to perform such studies (theme 1), major findings, the value of a user-driven approach, the importance of a well-balanced matching between the system functionalities and the users’ needs (theme 2), as well as the reported problems faced by each study team (theme 3). Therefore, this section will discuss the implications raised through the lens of each theme reported in this study.

By examining the research frameworks of studies included in this review, it clearly emerges that the use of ECAs deserves (1) a more sophisticated study design and (2) proof of the efficacy of the approach, as in any other technology designed for people with dementia. The key to managing both demands is to directly involve older adults with dementia in the design of services dedicated to them. The early engagement of users from the outset and across all stages of the development cycle is relevant for people affected by dementia, since they progressively lose the ability to generalize between past and present experiences or to modify cognitive representations. For this reason, familiarity with the technology to be used is to be firmly considered and needs to be carefully planned. Indeed, while healthy adults are easily able to manage routine changes such as introducing a new device into their home environment, for older adults with dementia, these novelties can become extremely distressing and disorientating. Moreover, different limitations can overburden users: limitations in knowledge and understanding of the technology and limitations in communication between the user and the technology. As discussed in themes 1 and 2, the use of a common television or computer screen and the possibility of using verbal response as an input/output mode enables a more natural way of interaction. This is a valuable benefit for people with mild and moderate dementia. According to Kaplan and Kaplan [52], familiarity is the relationship between an individual and something that this individual has had considerable experience with. The experience leads to the development of an internal model on how one expects something to work. Some of the included studies [15,46,47] revealed that less familiarity (a low experience with new technologies) and greater dementia severity will almost certainly lead to greater difficulty in accessing technology and inevitable intervention of the caregiver who will have to spend additional time to teach and support persons with dementia in using a new tool. This is in line with the state of the art in this field [53-57]. In fact, familiarity helps in encouraging older adults to learn and understand how to interact with new technologies by using their existing knowledge. Moreover, considering that perceived difficulties become more pronounced as dementia severity increases, the ability of a system to adapt to the changing needs and capabilities of users will determine the successful implementation of this system in everyday use.

The concept of familiarity is not the only principle to follow in the field of designing ECAs for persons with dementia. In the last decade, we conceived the important shift to a model of care centered on the person, which broke the traditional disease-focused approach. Thanks to this new paradigm, care and support are seen as ways to prevent functional decline, frailty, and disability [58] and to create a multifunctional status (ie, intrinsic capacity) to follow up over time [59,60]. Therefore, when approaching technological solutions that enable older people to remain independent at home, it is decisive to embrace the same paradigm: a model of design that follows the same path of the model of care, giving value to the person’s functions and needs [61-65]. Just as it is important to disseminate a model of care centered on the patients and their needs [66-68], it would be desirable to have a model of design that allows the participation of end users to propose more customized and consequently, more effective solutions. The benefits of a personalized design will spill over to end users who will achieve a higher level of well-being because they will see their needs met and caregivers whose burden will lighten. These concepts also emerged from some of the included studies [15,45-47] discussed in the theme 2. According to de Jong et al [46], persons with dementia cannot be treated as a homogeneous group and not in the same way. There is no “one right way” to take care of them, and one tool that will “fit all” cannot be created. Wargnier et al [15] also recommended a planning of technological strategies that consider the interpersonal variability of dementia and its evolution in time for each person. Moreover, Konig et al [45] underscored the importance of understanding past and current identities of persons with impaired cognitive abilities in technology-based efforts to provide individualized care and to suggest participatory design so that personalized solutions can be provided and the quality of life can be enhanced.

This new paradigm emphasizes the power of self-determination over decisions that affect the individual’s body and mind. Therefore, the individual dignity and autonomy, which are the primary values and the fundamental rights of every human being, are restored. In this new vision, patients actively participate in clinical decisions outside the old schema of only being a sick person who needs to be treated. Nowadays, well-being is the goal of dementia care that offers individualized interventions and considers the person as a whole, thus considering individuals’ medical, cognitive, psychological, environmental, cultural, and social needs [69]. Such individualized intervention can be co-designed with the direct involvement of patients. Co-designing creates a common knowledge base among designers, patients, and other stakeholders on the quality of life, pains, and gains and on how to support the remaining capabilities of persons with dementia [70]. The value of co-design is well recognized in all the studies reported in this narrative review. These considerations suggest that the field of new technologies such as ECAs needs to synchronize and harmonize knowledge, efforts, and challenges in the dementia care field and the new person-centered paradigm. For example, the respect of the principle of familiarity can promote the major involvement of people with dementia in the design of artifacts from the initial stage of the development process. Moreover, this engagement can also provide a sense of continuity for them, facilitating long-term use and acceptance of assistive devices along the disease’s progression. At the same time, usage continuity could open up the possibility of recruiting bigger samples of patients for enrollment in high-quality scientific research frameworks, thereby overcoming the limitations reported in the theme 3.

Another instance of harmonization is to focus on personhood and needs by clarifying what functionality and attributes are important in the new products for target users, what motivates them to use a product, what factors would hinder a positive user experience with a proposed product, and to conceptualize how parts of their lives could be improved by technology. Across the 3 themes analyzed in this review, the user experience of ECAs could be improved firstly by responding to the changing needs of people with dementia. This matching will enable technologies that better support the quality of life of people with dementia. This is particularly highlighted in the studies with more advanced ECAs such as Louise [15,44] and Anne [46,47]. Moreover, as predominantly reported in the 7 research studies, technologies should be able to adapt to the reserved skills of people with dementia without discouraging people with dementia from engagement; therefore, the natural modality of interaction by voice and the use of common screen devices are significant features to enable positive user experience. Additionally, as argued in themes 1 and 3, features such as interaction paradigm and prompting style are seen as the main components that can be personalized and used for matching the needs and capabilities of users, thereby improving the user experience. To date, the predominant use of technological solutions for safety and security [22] needs to be overcome by embracing a new paradigm that offers innovation supporting higher-level needs such as belonging, self-esteem, identity, and self-actualization [6]. The use of ECAs could be the future response to these higher-level needs and the management of everyday life across the disease’s journey. In any case, this approach seeks coordination between multidisciplinary teams composed of research elements, technology developers, health care communities, formal and informal caregivers, and primary users [6] as the core of the user-driven approach [51]. As reported in this narrative review, the significant valence of the user-centered design as well as the iterative measurements of the usability and acceptance rate are still milestones to achieve during the research and devolvement process of technologies for people with dementia.

### Comparison With Prior Works and Limitations

To the best of our knowledge, no other narrative reviews are reported in the literature regarding the research frameworks used to study the interaction between persons with dementia and ECAs and between the mapped outcomes and barriers. Despite this positive aspect, there are some limitations to this review. Data sources were drawn from only 4 databases (ie, Scopus, Web of Science, PubMed, and Embase) and accessed only during a specific period of time (July 2020). The choice of using specific phrases to target the population (“patient with dementia” OR “people with dementia” OR “person with dementia”) and the specific technology-based intervention (“virtual agent” OR “personal virtual assistant” OR “virtual companion” OR “embodied conversational agent”) could have omitted some results from the search. It is possible that other literary sources were available in other unselected databases. However, well-known and broad-spectrum databases were used in this review. Moreover, we collected a relatively small sample of studies and excluded non-English language studies. Therefore, even if the 7 studies included in this paper were homogeneous in terms of their qualitative research design and their meeting our inclusion criteria, this may have created some biases. In addition, some authors of this review are co-authors in 2 of the reported studies [46,47]. Despite these limitations, our study offers several research directions, which may take the existing debates to the next level.

### Future of ECAs

This review mapped the actual use of ECAs in the research field of dementia. The readiness level of this specific technology-based intervention grew across the years, shifting from to be initially displayed on a standard television set [24] and computer screen [15,27,44,45] to mobile standalone solutions [46,47]. This led to important achievements in the visual representation as well as in the conversational abilities of the ECAs that technologically could be seen as the foundation for advanced applications in the near future as nontherapeutic interventions to assist individuals with dementia. However, despite these promising improvements over 12 years, it remains difficult to prove that ECAs are effective to mimic interpersonal communication when interacting with users and safe to use in the care practice. Technological advances in the embodiment, content, communication modality, and strategy are not indeed the only axes of improvement since there is still to discover how preferences regarding the appearance, animation, and personalized features can influence user acceptance and efficacy of the intervention. The scarcity of evaluation and implementation phase studies underlined the necessity for further research with larger sample sizes, suitable control groups, and clinical populations but the emerging interest on the field is a gaining advancement [71,72]. Anyway, possible steps forward for the use of such systems in health care delivery can be seen in integrated platform services. For example, telemedicine, ambient intelligence, and machine learning systems can be improved through conversational agents especially in the area of health counselling, coaching, psychotherapy, and self-monitoring. Additionally, interactions between virtual agents and advanced robotics is a new design challenge [73] that could embody ECAs in social robots enforcing the attention, facial expressions, and tone of voice of future human-like robots.

### Conclusions

This narrative review summarizes the current research on ECAs for patients with dementia. Technologically, these artificial characters are very interesting and the mapped studies shared promising results in terms of engagement of patients. Unfortunately, until now, it has been difficult to prove that ECAs are effective and more efforts need to be spent to achieve to this evidence. Therefore, our thematic analysis reported on 3 main themes, namely, the research frameworks used to gather users’ perspectives on ECAs (theme 1), the valuable insights shared by the 7 studies as well as the value of user involvement in the development phases and the challenge of matching the system functionalities with users’ needs (theme 2), and the main methodological and technical problems faced by each study team (theme 3). It emerged that this specific field of research is novel and poorly discussed in the scientific community, but possible steps forward for the use of such systems in health care delivery are predictable. Moreover, analyzing the main metaphors across the studies, our work underscored the challenge to synchronize and harmonize knowledge, efforts, and challenges within the dementia care field and its person-centered paradigm. This can be effectively possible by adopting the well-known but still little used user-centered design [51] approach, which standardizes the compelling multidisciplinary vision of research and development of innovative technologies. The challenge is therefore to enable strict collaboration between interdisciplinary research networks, medical scientists, technology developers, patients, and their formal and informal caregivers.

## Appendix

Multimedia Appendix 1 PRISMA checklist.

## Abbreviations

ECA: embodied conversational agent
